# Unraveling Hierarchical B‐Site Ordering: Mechanism of Microwave Dielectric Enhancement in Complex Perovskite Oxide Ceramics

**DOI:** 10.1002/advs.76785

**Published:** 2026-07-29

**Authors:** Qingqiao Fu, Hui Gu, Hanbin Gao, Pianpian Ma, Xiangming Chen, Juanjuan Xing, Qiang Zheng

**Affiliations:** ^1^ School of Materials Science and Engineering Shanghai University Shanghai China; ^2^ Center for High Pressure Science & Technology Advanced Research Beijing China; ^3^ CAS Key Laboratory of Standardization and Measurement for Nanotechnology National Center for Nanoscience and Technology Beijing China; ^4^ School of Materials Science and Engineering Zhejiang Sci‐Tech University Hangzhou China; ^5^ School of Materials Science and Engineering Zhejiang University Hangzhou China

**Keywords:** complex perovskite oxides, hierarchical ordering, microwave dielectric, multiscale characterization

## Abstract

Complex perovskite oxides with mixed cation occupancy are important class of functional materials, offering tunability for microwave dielectric properties, ferroelectricity, and magnetism. In despite of their significant utility, the correlation between different hierarchical levels of cation ordering, ranging from macroscopic domains to atomic‐scale clusters, remains elusive, impeding optimization and rational design of high‐performance ceramics. Here, a comprehensive multiscale characterization route is established to quantify ordering in a model 1:2 B‐site‐ordered microwave dielectric system, Ba[(Co_0.6‐_ *_x_*_/2_Zn_0.4‐_ *_x_*_/2_Mg*_x_*)_1/3_Nb_2/3_]O_3_. By integrating multiscale characterization techniques, the evolution of ordering is tracked from the micrometer to the sub‐angstrom level. Significant lattice strain is observed within the ordered domains, which is strongly correlated with the specific arrangement of B‐site cations. The concentration of oxygen vacancies increases with increasing annealing temperature. Local chemical fluctuations, specifically the compositional variability of multiple cations and Mg disorder, are also identified. Collectively, these three factors disrupt the translational symmetry of the lattice. They act as exceptionally strong phonon scattering centers that significantly shorten the phonon lifetime, ultimately affecting the performance. This work provides a possible path for future optimization and design of dielectric properties through manipulating chemical ordering, point defects, and lattice strain within ordered domains in complex perovskite microwave dielectric ceramics systems.

## Graphical abstract

This study maps out the hierarchical ordering in 1:2 B‐site complex perovskite ceramics using a comprehensive multiscale characterization route. It turns out that intradomain strain, oxygen vacancies, and Mg disorder within the ordered domains act as strong phonon scatterers. This scattering shortens phonon lifetime and degrades the microwave dielectric.

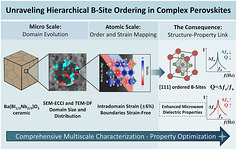

## 1 Introduction

Perovskite oxides (ABO_3_), exhibit exceptional compositional flexibility at both the A and B sites, resulting in diverse structural types and novel physical properties that have sparked significant interest in condensed matter physics and materials science [1, 2, 3]. The occupation of various cations at A and B sites, either in an ordered or disordered manner, plays a pivotal role in influencing and controlling properties of materials, such as magnetism, ferroelectricity, and thermoelectricity [4, 5, 6]. Consequently, elucidating the distribution of cations and the related microstructural characteristics is critical for designing new structures, optimizing functional properties, and practical applications. However, previous studies have predominantly focused on the impact of ordered versus disordered structures on specific properties. There remains a lack of quantitative insight into the underlying mechanism governing local microstructure, composition, and physical properties from a multiscale perspective. Furthermore, the correlation between different levels of ordering, ranging from local cations arrangements to the macroscopic volume fraction of ordered domains, is complicated and remains poorly understood. This hinders the precise optimization of processing conditions and the tailored design of relevant physical properties. Therefore, selecting a representative perovskite oxide material, with different types of cations occupying the same crystallographic site, to systematically quantify the correlation between ordering degrees at various scales is essential for understanding the fundamental nature of ordering and for designing multi‐component complex perovskite oxide materials.

B‐site‐ordered complex perovskite ceramics, A(B^I^ *_x_* B^II^ *_y_*)O_3_ (where x:y = 1:1, 1:2, 1:3), are important dielectric materials with significant applications as resonators and filters in microwave communication systems [7, 8]. Among these, 1:2 the B‐site‐ordered complex perovskite ceramics, Ba(B^I^_1/3_B^II^_2/3_)O_3_ (where B^I^ is typically Mg^2+^, Ca^2+^, Sr^2+^, Mn^2+^, Co^2+^, Ni^2+^, Cu^2+^, Zn^2+^; and B^II^ is usually Nb^5+^, Ta^5+^), have been attracting significant attention due to their outstanding microwave dielectric properties [9, 10]. These structures feature a 1:2 ordered layering of B^I^ and B^II^ cations (.B^I^B^II^B^II^.) along the [111] direction (ordering vector 1/3[111]^*^), yielding a hexagonal superstructure that significantly enhances the dielectric response [11, 12, 13]. Recently, researchers have extensively studied enhancing microwave dielectric properties of such 1:2 B‐site‐ordered complex perovskite ceramics to broaden their applications, involving optimizing processes, designing new compositions, and exploring dielectric polarization [14]. However, few studies have explored the underlying mechanism and optimized performances for these materials from the aspect of controlling or designing different levels of ordering degree [15, 16, 17], limiting the comprehensive understanding of key factors that dominate the properties like dielectric constants *ε_r_*, quality factor *Qf* (the inverse of the dielectric loss) and temperature coefficient of resonance frequency *τ_f_*.

Currently, studies on ordering degree in 1:2 B‐site‐ordered complex perovskite ceramics have still been limited in the so‐called ordered domains. The assessment of ordering degree in these ceramics relies heavily on X‐ray diffraction (XRD). This method estimates the ordering degree (S) by comparing the intensity ratio of superlattice peaks to fundamental peaks against theoretical values, as expressed as $S=\frac{\sqrt{{\left(I_{100}/I_{110,102}\right)}_{observed}}}{\sqrt{{\left(I_{100}/I_{110,102}\right)}_{theoretical}}}\;$ [17, 18, 19]. However, XRD provides only a volume‐averaged structure, failing to reveal spatial uniformity or local variations. Additionally, while transmission electron microscopy (TEM) diffraction contrast imaging has been used to visualize domain size and distribution [20, 21]. it often lacks precise chemical information regarding specific B‐site distributions. Moreover, atomic‐scale features, including elemental arrangement within domains, and local lattice strain, remain unexplored, despite their critical influence on microwave dielectric property. Therefore, it is quite urgent to establish a multiscale analysis methodology, from the micrometer to the sub‐angstrom level, to enable precise characterization and quantitative analysis of ordering degree and domains in 1:2 B‐site‐ordered complex perovskite ceramics and to systematically establish their microstructure—property correlation mechanism.

In this study, we selected a series of high‐performance 1:2 B‐site‐ordered complex perovskite ceramics Ba(B^I^_1/3_Nb_2/3_)O_3_ as a model system to develop a comprehensive quantitative method for investigating different levels of ordering degrees and domain structures. We elucidate the effects of B^I^‐site composition and annealing temperature on microstructural features, deduce their formation and evolution processes, and establish the competitive relationship between order and disorder. Furthermore, we clarify the underlying mechanisms linking multiscale microstructure to physical properties, providing a framework for the future design of complex perovskite oxide materials.

## 2 Results

### 2.1 Macroscopic and Nanoscale Assessment of Ordering Degrees

**FIGURE 1:**
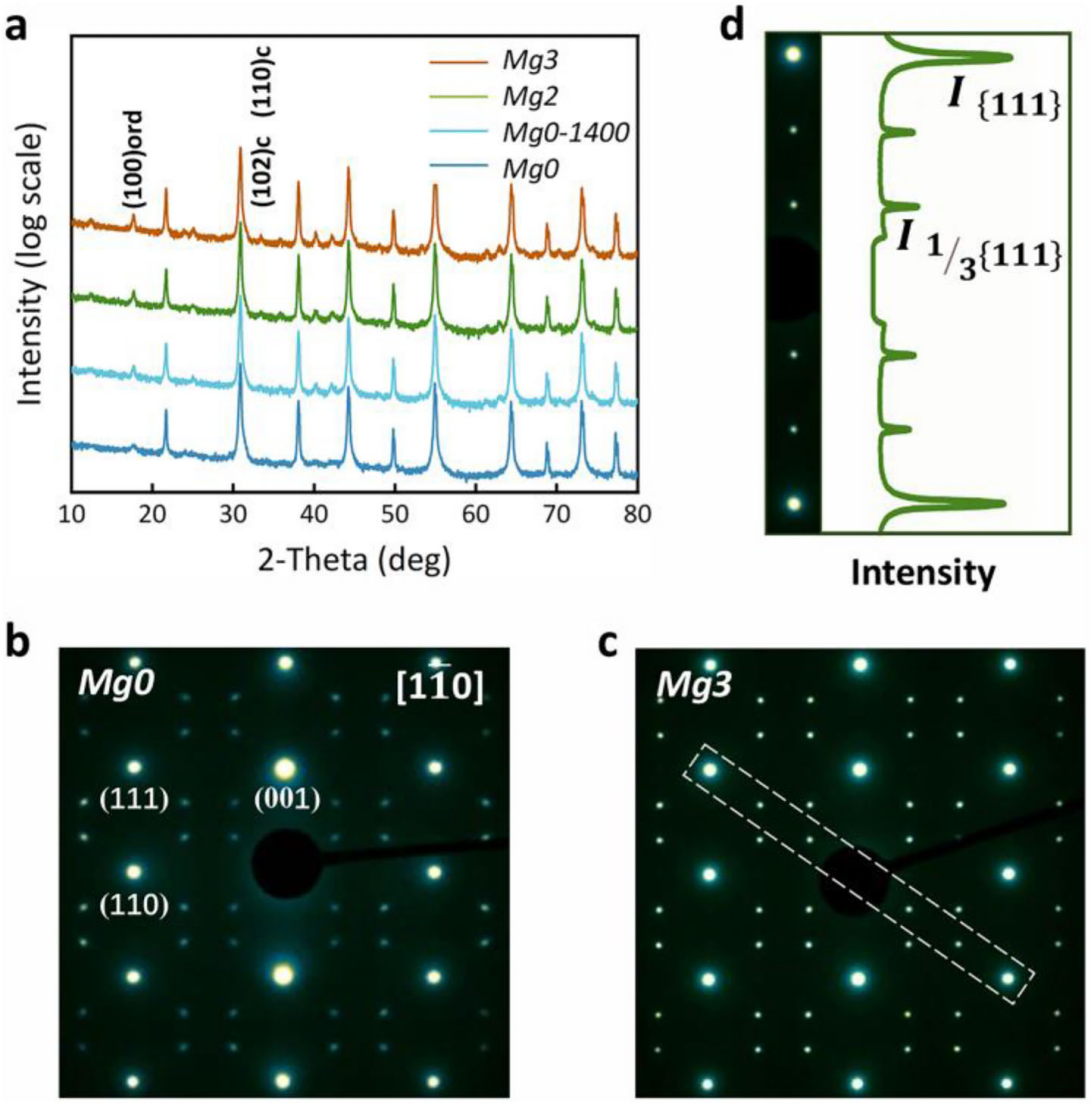
Structural characterization of cation ordering. (a) XRD patterns of Mg0, Mg0‐1400, Mg2, and Mg3 ceramics. (100)_ord_ superstructure reflection is labelled and appears in all four patterns. All XRD data were obtained from reference [16]. (b,c) SAED patterns of Mg0 and Mg3 ceramics viewed along [1$\bar{1}$0]_cubic_ zone axis. While ±1/3{111} superlattice reflections are observed in both patterns, they appear significantly weaker in Mg0 compared to Mg3. (d) Intensity extracted along the reflections indicated by the dashed rectangular region in the SAED pattern of the Mg3 ceramic in (c).

Traditionally, the ordering degree in 1:2 B‐site‐ordered perovskite oxide materials is quantified via XRD by comparing the observed intensity ratio of the superlattice diffraction peak to the strongest diffraction peak against the theoretical ratio for a fully ordered structure, as expressed as $S=\frac{\sqrt{{\left(I_{100}/I_{110,102}\right)}_{observed}}}{\sqrt{{\left(I_{100}/I_{110,102}\right)}_{theoretical}}}\;$ [17, 18, 19]. As shown in Figure 1a, the relative intensity of superlattice diffraction peaks in the XRD patterns of Ba[(Co_0.6‐_ *_x_*_/2_Zn_0.4‐_ *_x_*_/2_Mg*_x_*)_1/3_Nb_2/3_]O_3_ (*x* = 0, 0.2, and 0.3) ceramics increases with the increasing of Mg concentrations. The calculated ordering degree for Mg0, Mg2, and Mg3 were approximately 53.6%, 89.3%, and 90.1% respectively. Moreover, increasing the annealing temperature can lead to a further enhancement in the ordering degree of the ceramics, with approximate values of around 86.0%, 94.1%, and 94.6% for Mg0‐1400, Mg2‐1400, and Mg3‐1400 ceramics, respectively (Table 1). These original XRD data were obtained from reference [16]. However, it's worth to noting that this method only provides an “averaged” ordering degree of the material structure and does not reveal the uniformity of the ordering degree and their spatial distribution.

**TABLE 1:** Parameters of sintering, microstructure and properties of the Ba[(Co_0.6‐x/2_Zn_0.4‐x/2_Mg_x_)_1/3_Nb_2/3_]O_3_ ceramics (*x* = 0, 0.2, 0.3). Values of the dielectric constants(*ε_r_*), quality factor(*Qf*) and temperature coefficient of resonance frequency (*τ_f_*), along with the XRD data, are based on reference [16].

	T_s_ (°C/3h)	T_a_ (°C/12h)	Grain size (µm)	Domain size (nm)			Order degree (%)			*ε_r_* [16]	*Qf* [16] (GHz×1000)	*τ_f_* [16] (ppm/°C)
				XRD	SEM	TEM	XRD [16]	TEM	SAED			
**Mg0**	1475	—	5.4 ± 2.7	11	14 ± 4	5 ± 2	53.6	58.2	48.3	35.7	44.5	7.2
**Mg0‐1400**	1475	1400	5.5 ± 2.7	30	37 ± 7	31 ± 5	86.0	86.9	88.3	35.2	75.0	4.4
**Mg2**	1525	—	7.2 ± 3.6	21	51 ± 7	40 ± 6	89.3	90.7	90.1	34.1	86.4	8.3
**Mg2‐1400**	1525	1400	7.3 ± 3.2	34	68 ± 20	55 ± 14	94.1	94.9	90.3	33.7	114.4	7.6
**Mg2‐1450**	1525	1450	7.3 ± 3.3	—	112 ± 21	78 ± 14	—	97.3	90.3	33.5	113.2	7.0
**Mg2‐1475**	1525	1475	7.2 ± 3.8	—	127 ± 28	93 ± 19	—	99.9	90.3	33.1	111.0	6.3
**Mg3**	1550	—	8.4 ± 3.5	24	72 ± 11	55 ± 5	90.1	92.9	90.3	33.7	93.8	9.6
**Mg3‐1400**	1550	1400	8.3 ± 3.2	33	83 ± 20	67 ± 15	94.6	96.8	90.7	33.3	117.2	8.6
**Mg3‐1450**	1550	1450	8.3 ± 3.1	—	127 ± 28	97 ± 20	—	98.0	90.7	33.1	115.8	7.8
**Mg3‐1475**	1550	1475	8.3 ± 3.3	—	150 ± 35	127 ± 31	—	99.9	90.7	33.0	114.1	6.9

Selected Area Electron Diffraction (SAED) offers a method to assess the ordering degree at the nanoscale. Figure 1b displays weak ±1/3{111} superlattice reflections in the Mg0 ceramic. The intensity of these superlattice reflections increases with both Mg concentrations (Figure 1c) and annealing temperatures. Analogous to XRD, the ordering degree can be quantified through the intensity ratio of the ±1/3{111}_cubic_‐type superlattice reflection to the {111}_cubic_ main reflections in SAED​, which is then compared to the theoretical value for complete ordered (Figure 1d), as expressed as $S\;=\frac{\sqrt{{\left(I_{1/3\left\{111\right\}}/I_{\left\{111\right\}}\right)}_{observed}}}{\sqrt{{\left(I_{1/3\left\{111\right\}}/I_{\left\{111\right\}}\right)}_{theoretical}}}\;$. Compared to XRD technique, this method can show the information on nanoscale ordering degree and their spatial distribution. However, it's noteworthy that the calculated values of ordering degree from SAED patterns among these ceramics were consistently lower, never exceeding 91% (Table 1).

### 2.2 Microstructural Evolution and Domain Distribution

SEM can reveal ordering degree at submicron scales. SEM analysis (Figure S1) reveals a dense microstructure for all compositions. The average grain size increases from 5.4 µm (Mg0) to 8.4 µm (Mg3) with more Mg concentrations, attributed to the higher sintering temperatures required for Mg‐rich compositions. Specifically, 10% more Mg^2+^ concentrations correspond to 25 °C increasing in the optimum sintering temperature of the ceramics [16]. However, annealing temperatures have a negligible impact on grain sizes, as the detailed grain size statistics is listed in Table 1. Since all grain sizes exceed 5 µm, the volume fraction of grain boundaries is minimal, and their contribution to high‐frequency polarization loss is negligible [22]. Furthermore, these ceramics present a homogeneous composition without secondary phase, as confirmed by EDS elemental mapping and exemplified by the microstructure of the sample with *x* = 0.3 (Mg3) shown in Figure S2.

**FIGURE 2:**
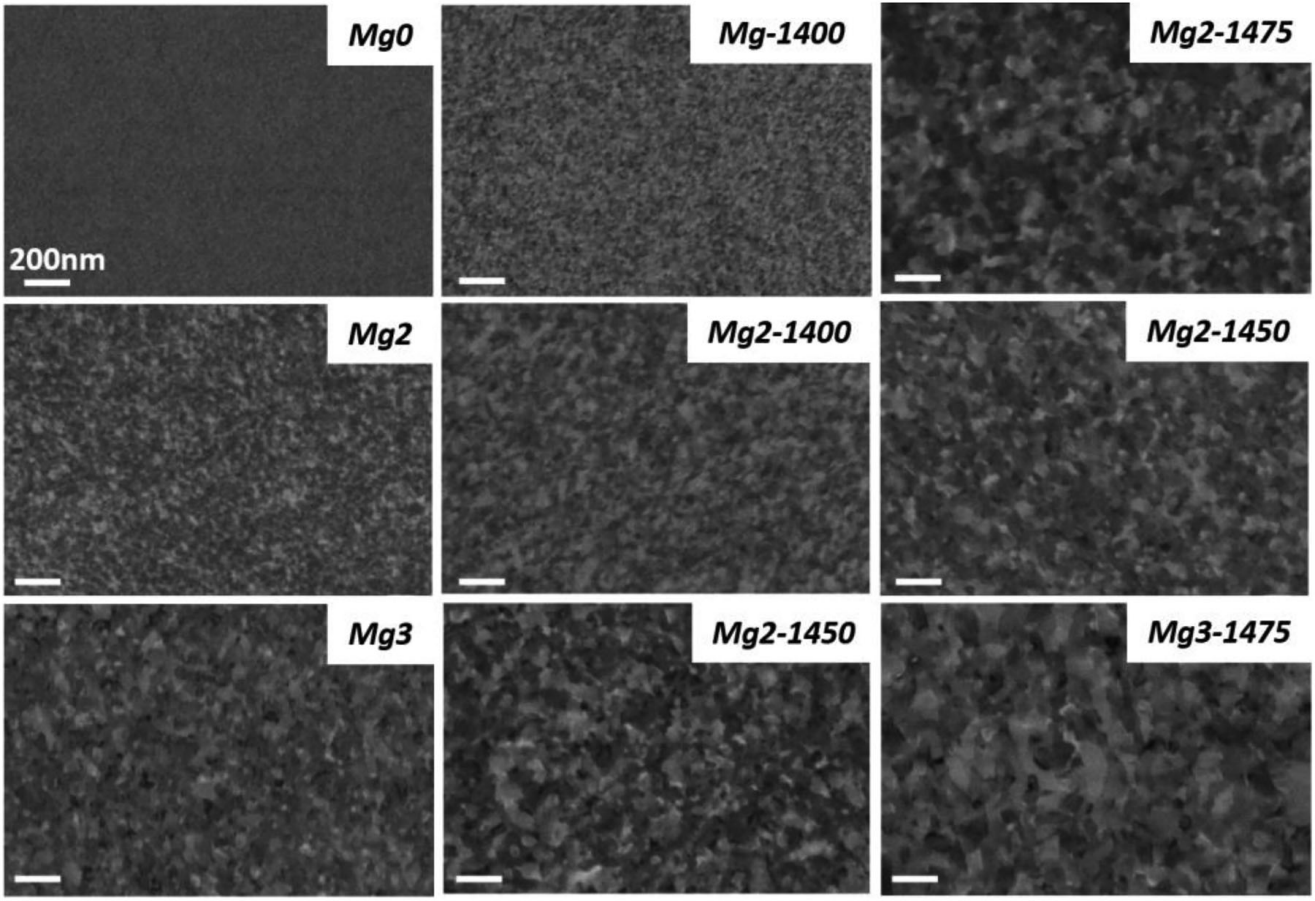
Microstructural evolution and domain structures. SEM‐ECCI images of ceramics showing that ordered domains gradually grow with increasing Mg concentration and annealing temperature. Note that domains in Mg0 are not visible owing to resolution limitation.

To comprehensively and systematically characterize domains, their distributions and the ordering degree in each ceramic, ECCI is thereby employed, which has emerged as a pivotal method within SEM for analyzing domains at submicron scales. It relies on backscattered‐electron channeling, whose contrast is acutely sensitive to local crystal orientation and lattice perfection [23, 24, 25]. This approach allows for efficient and rapid characterization of larger regions, making it particularly suitable for quantification on sizes and distributions of domains at the submicron scale. It is visually apparent that the average domain sizes of Mg0, Mg2, and Mg3 ceramics are approximately 14 nm, 51 nm, and 72 nm respectively, indicating an increasing tendency of average domain size with increasing Mg concentration (Figure 2). Furthermore, the average domain size rapidly increases after annealing (Figure 2). For example, domains in Mg3 grew to 83 nm, 127 nm, and 150 nm after annealing at 1400 °C, 1450 °C, and 1475 °C, respectively (Table 1).

### 2.3 Quantitative Dark‐Field TEM Analysis

**FIGURE 3:**
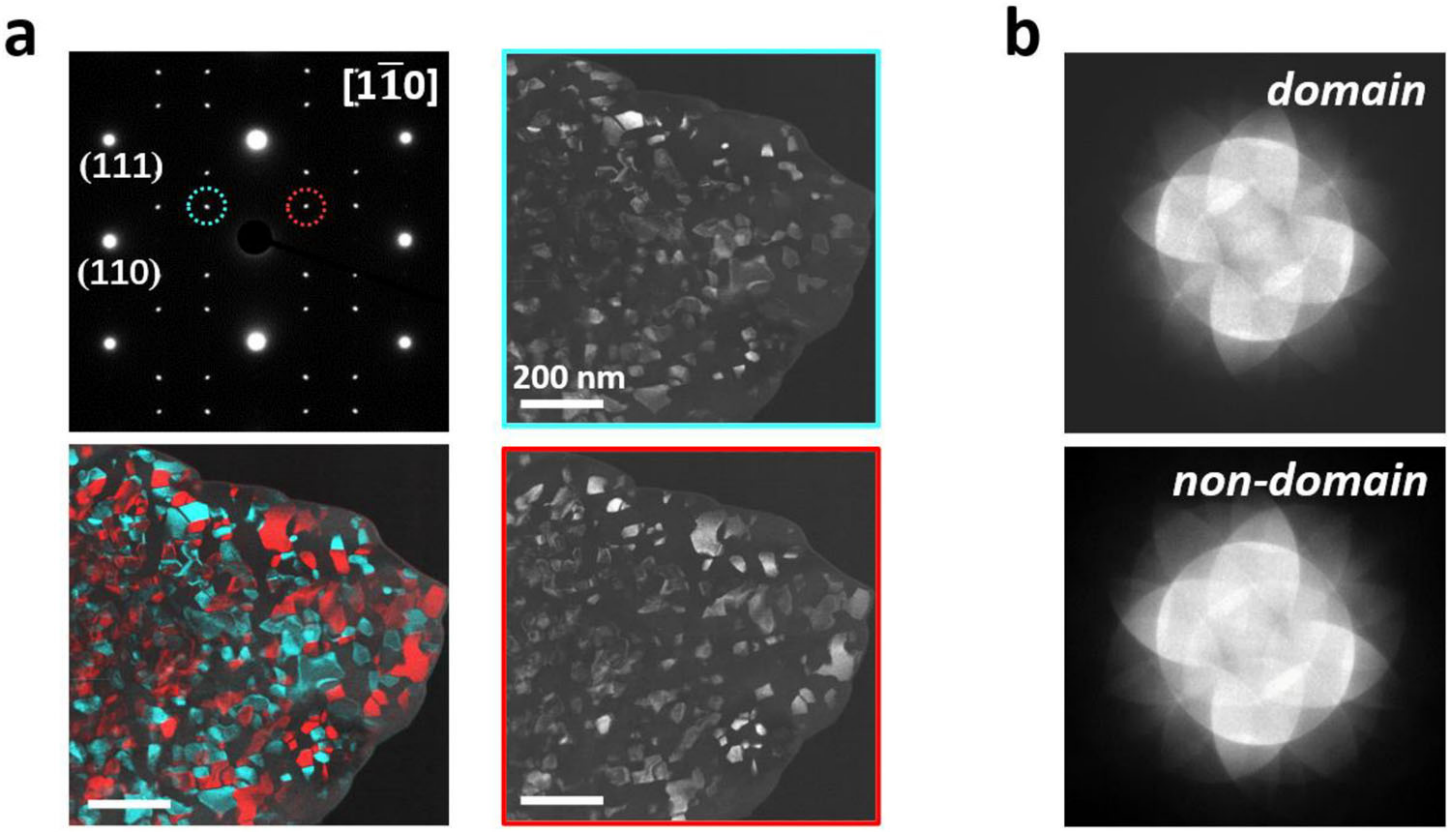
Spatial distribution of ordered domain. (a) Illustration of the domain maps of Mg2‐1400: superstructure reflection 1/3(111) (masked by cyan circle) and 1/3($\bar{1}\bar{1}$1) (masked by red circle) in the SAED pattern of the 1:2 B‐site‐ordered complex perovskite (viewed along [1$\bar{1}$0]_cubic_ zone axis) were respectively selected for TEM‐DF imaging. The resulting TEM‐DF images are depicted in the right panels. These two TEM‐DF images are overlaid to construct a composite distribution map of domains with varying orientations (lower left panel). (b) CBED images of ordered regions and the regions lacking ordered features in Mg3‐1475.

TEM dark‐field (DF) imaging provides precise nanoscale information on domain structures. Owing to the diverse orientations of these domains, each corresponds to distinct superlattice spots in the SAED pattern. For example, in the SAED pattern of the Mg2‐1400 ceramics along the zone axis of the [1$\bar{1}$0]_cubic_ (Figure 3a), superlattice reflections of (*h*±1/3, *k*±1/3, *l*±1/3) appear, originating from 1:2 B‐site ordering and corresponding to a tripling of the unit cell along a single unique <111> direction [11, 12, 13]. By selecting a specific superlattice diffraction spot, a corresponding TEM‐DF image can be acquired, where bright regions denote domains exhibiting that particular ordering. Overlaying a series of TEM‐DF images from the same regions yields a spatial distribution map of domains with different ordering orientations, as shown in Figure 3a. In this map, dark regions contain both disordered domains and domains aligned parallel to the electron beam.

**FIGURE 4:**
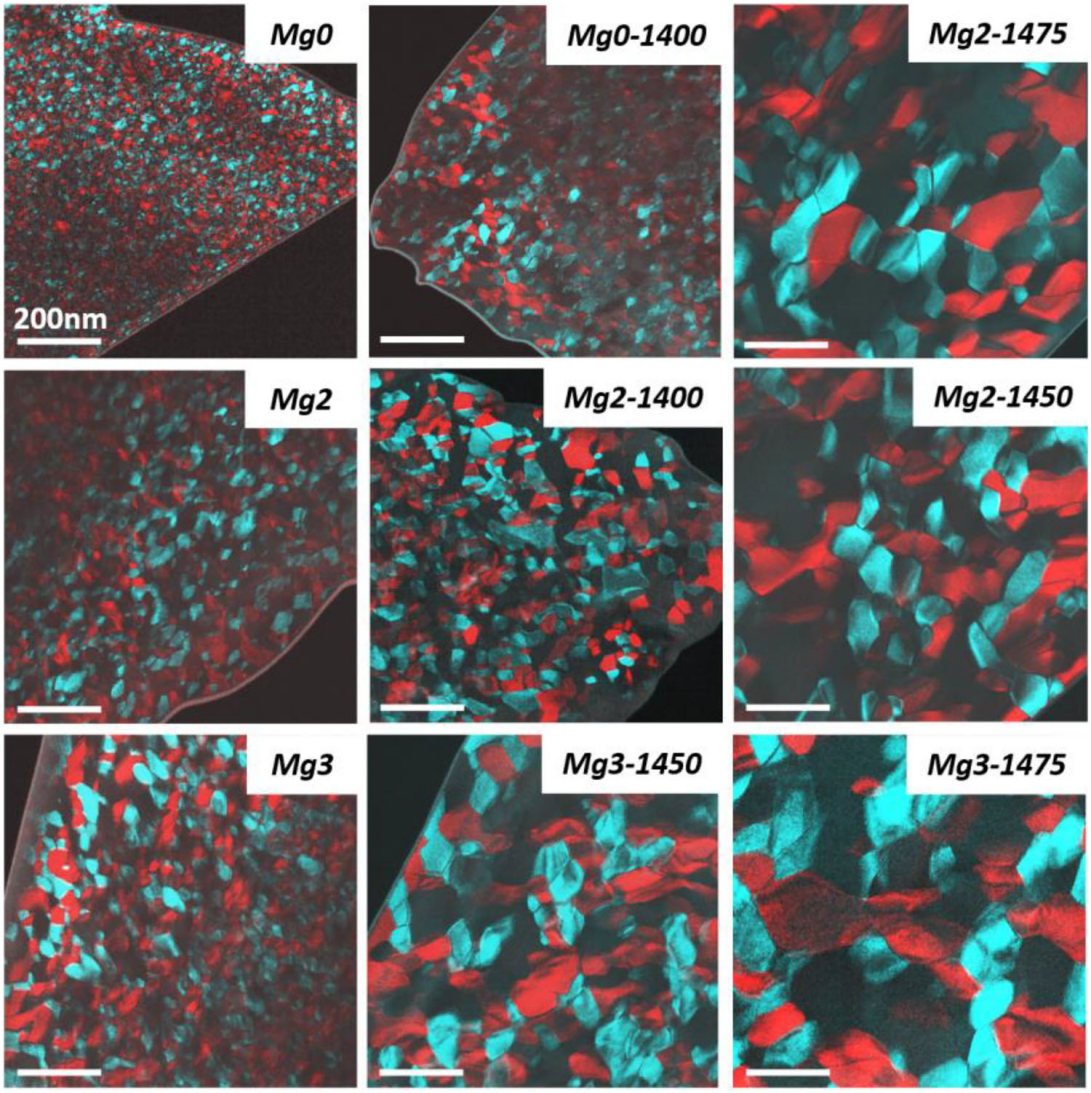
Quantitative dark‐field TEM analysis. Overlaid false‐color TEM‐DF images of different ceramics, characterized using the method described in Figure 3a, showing that ordered domains in different directions grow with increasing Mg concentration and annealing temperature.

Using TEM‐DF imaging (Figure 4), the average domain sizes for Mg0, Mg2, and Mg3 ceramics were measured to be approximately 5 nm, 40 nm, and 55 nm, respectively. As the annealing temperature increases, the average domain sizes for Mg3‐1400, Mg3‐1450, and Mg3‐1475 samples increase to 67 nm, 97 nm, and 127 nm, respectively (Table 1). These results exhibit trends similar to those observed in SEM‐ECCI images. However, compared with SEM‐ECCI, TEM‐DF imaging offers excellent capability for distinguishing domains with varying ordering orientations and outlining domain boundaries. Furthermore, TEM‐EDS elemental mapping of the Mg3 ceramic (Figure S3) confirms identical chemical compositions across domains with distinct ordering orientations, thereby ruling out composition‐driven domain formation.

Additionally, the convergent beam electron diffraction (CBED) technique provides three‐dimensional structural information of the analyzed region [26, 27]. To further clarify the dark regions in TEM‐DF images and determine whether these regions are ordered or disordered, CBED analyses were performed to compare domains showing ordered patterns along the [1$\bar{1}$0]_cubic_ zone axis and regions lacking visible ordering features (but potentially exhibiting ordering parallel to the electron beam). To correctly interpret these CBED observations, it is necessary to understand the crystallographic symmetry constraints governing domain variants in this material. Based on crystallographic symmetry, in complex perovskite materials with B‐site 1:2 ordering, the domains develop along the <111> directions of the cubic lattice. In this crystal system, there are eight equivalents <111> directions, corresponding to four domain variants. When observed via transmission electron microscopy along the [1$\bar{1}$0]_cubic_ zone axis, the superlattice diffraction spots of four variants, [111], [11$\bar{1}$], [$\bar{1}\bar{1}\bar{1}$] and [$\bar{1}\bar{1}$1] are clearly visible, whereas those of the other four variants, [1$\bar{1}$1], [1$\bar{1}\bar{1}$], [$\bar{1}$1$\bar{1}$] and [$\bar{1}$11] remain invisible. According to crystallographic symmetry and the principle of equal thermodynamic probability, these eight domain variants should possess equal volume fractions within the sample. Guided by this symmetry analysis, we return to the CBED results to examine the regions lacking visible ordering features. As shown in Figure 3b, regions without visible ordering indeed display weak ordered symmetry, indicating that these areas contain ordering parallel to the electron beam. Therefore, the combined interpretation from SAED, TEM‐DF imaging, and CBED analysis enables comprehensive understanding of domain distribution and ordering characteristics.

Moreover, TEM‐DF techniques can be utilized to quantify the local ordering degree and the proportion of different domain orientations. This is defined as the ratio of the average total area of ordered domains to the total image area within a grain. It should be noted that only about 50% of domains are visible in TEM‐DF images along the [1$\bar{1}$0]_cubic_ zone axis, which has been corroborated by previous modeling studies [11]. while the remaining 50% are oriented parallel to the same axis, consistent with the CBED observations discussed above. Therefore, the measured area ratio of ordered regions in TEM‐DF images must be multiplied by two to estimate the overall ordering degree within the whole grain. Based on this, the ordering degrees for Mg0, Mg2, and Mg3 ceramics were calculated to be approximately 58.2%, 90.7%, and 92.9%, respectively, revealing a clear trend of increasing ordering with higher Mg concentrations. Likewise, raising the annealing temperature can enhance the ordering degree for Mg0‐1400, Mg2‐1400, and Mg3‐1400, of which the degrees of ordering are 86.9%, 94.9%, and 96.8%, respectively.

### 2.4 Physical Principles and Complementary Roles

XRD, ECCI, SAED, and TEM‐DF all show consistent trends in the evaluated ordering degree, though with quantitative discrepancies. Specifically, relative to XRD, SAED underestimates and TEM‐DF overestimates the ordering degree. This discrepancy can be understood from their underlying physical principle. XRD analysis is generally interpreted within the kinematical diffraction approximation, where the diffracted intensity scales with the squared modulus of the structure factor and is only weakly affected by multiple scattering. Thus, the ordering degrees derived from structural refinement serve as a reliable absolute benchmark. In contrast, SAED and TEM‐DF are inherently compromised by dynamical diffraction and local thickness variations. As the electron beam propagates through the crystal, multiple scattering systematically attenuates or redistributes superlattice intensities. Moreover, TEM‐DF domain contrast depends heavily on orientation and local diffraction conditions, preventing direct conversion into an absolute ordering degree.

Despite these differences, the techniques offer complementary insights rather than contradictory values across different scales. Our strategy is therefore to establish XRD as the definitive reference for average bulk ordering. SAED, with its known underestimation, is best suited for semi‑quantitative, localized analysis. TEM‐DF reveals the apparent volume fraction of ordered domains, ideal for tracking spatial distribution and morphological changes. ECCI, sensitive to local crystal orientation and lattice integrity, bridges the gap between macroscopic XRD and microscopic TEM. A comprehensive comparison of detection scales, principles, advantages, and limitations is provided in Table 2.

**TABLE 2:** Comparison of characterization methods for B‐site ordering in complex perovskite ceramics.

Method	Probed scale	Physical principle	Main advantages	Limitations
XRD (bulk‐averaged ordering parameter)	Bulk (mm‐scale)	Kinematical diffraction; Minimal multiple scattering	Reliable macroscopic reference; Quantitative	Cannot resolve local distribution; Insensitive to heterogeneity
ECCI (mesoscale channeling‐derived domain mapping)	Mesoscale (µm to mm‐scale)	Electron channeling effect; Contrast modulated by local orientation	Non‐destructive; Bridges macro‐XRD and micro‐TEM.	Requires damage‐free surface; Strong orientation dependence
SAED (local diffraction‑derived ordering indicator)	Local (sub‐µm to µm‐scale)	Dynamical diffraction; Strong multiple scattering	Direct visualization of superlattice spots; Local phase identification	Ordering degree underestimated; Semi‐quantitative
TEM‐DF (the volume fraction of ordered regions)	Local (nm‐scale)	Dark‐field imaging using superlattice reflection	Directly reveals domain size, morphology, and connectivity	Apparent fraction ≠ absolute ordering degree
HRTEM‐EDS (atomic‐scale cation‐distribution mapping)	Local (nm‐scale)	Atomic‐resolution imaging; Local elemental mapping by characteristic x‐ray signals	Directly identifies local B‐site cation arrangement and compositional segregation	Limited to localized regions; Sensitive to sample thickness and beam damage

## 3 Discussion

### 3.1 Atomic‐Scale Analysis of Domain Boundaries and Lattice Strain within Domains

**FIGURE 5:**
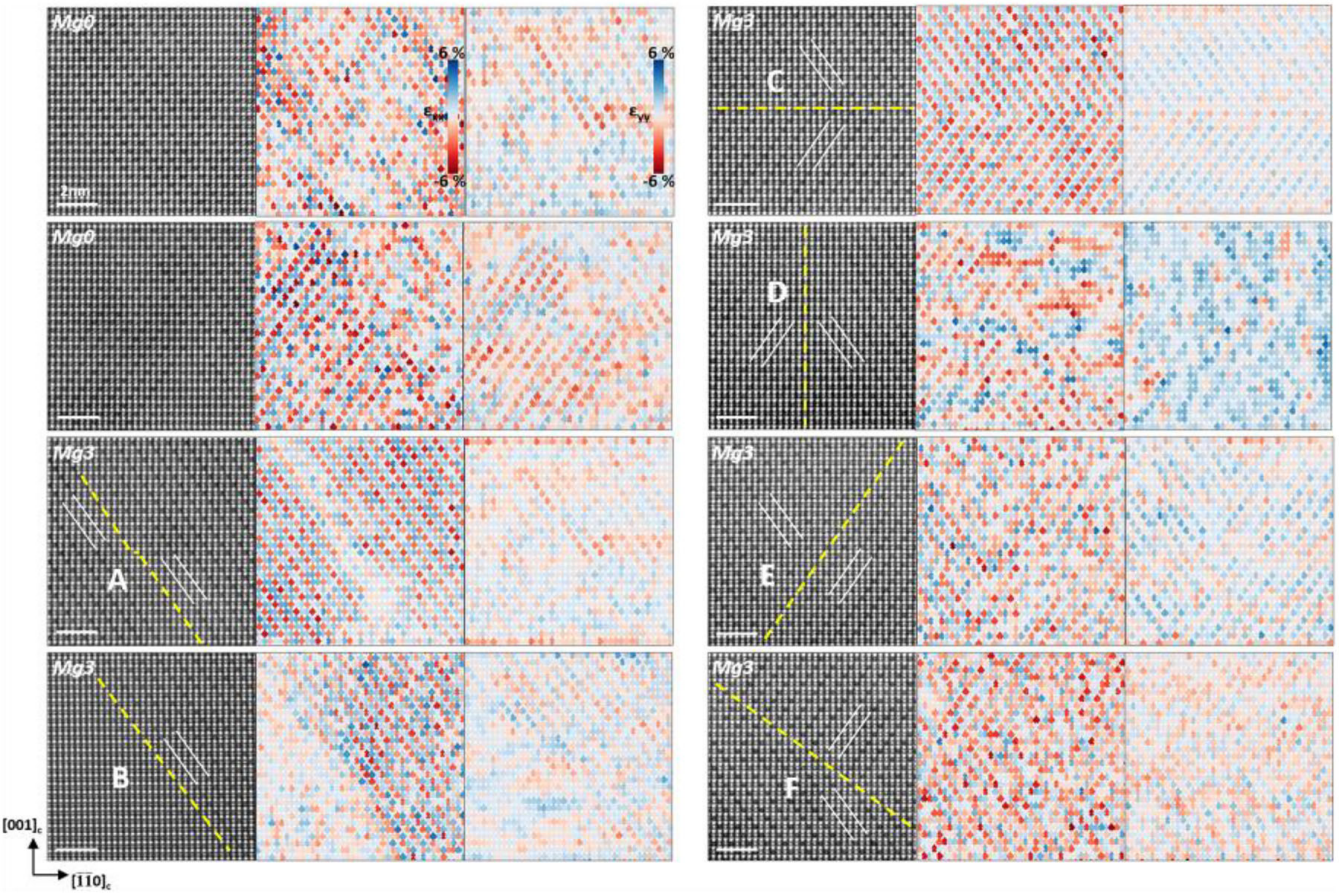
Atomic‐scale strain analysis within domains and at domain boundaries. HAADF‐STEM images along [1$\bar{1}$0]_cubic_ (left panels), and corresponding lattice strain maps along the x‐direction (middle panels) and y‐direction (right panels) across different types of domain boundaries. The analysis indicates that the boundaries exhibit continuous transitions and do not exhibit obvious strain. In contrast, the strain distribution within the domains is dominated by the 1:2 B‐site ordering, showing tensile strain on the B^I^‐site atoms and compressive strain on the B^II^‐site atoms, with maximum strain magnitudes reaching approximately 6% and ‐6% respectively.

Beyond the domain structure and ordering degree in Ba[(Co_0.6‐_ *_x_*_/2_Zn_0.4‐_ *_x_*_/2_Mg*_x_*)_1/3_Nb_2/3_]O_3_ ceramics, the atomic structure of domain boundaries and ordering degree within domains are essential for understanding the competitive relationship between order and disorder structure and the corresponding correlation between multiscale microstructure and properties. Figure 5 presents HAADF‐STEM images of ceramic. In Mg0 sample, domains are small (5‐7 layers) and isolated, with a low proportion. With increased Mg concentrations or annealing temperatures, domains coarsened rapidly and densely covered the entire grain, forming a significant number of domain boundaries. This is evidenced by the observation of six types of domain boundaries in the projection of zone axis [1$\bar{1}$0]_cubic_: A‐type boundaries with the same superlattice orientations, B‐type boundaries between visible and invisible domains, C‐type boundaries parallel to (001)_c_, D‐type boundaries parallel to (110)_c_, E‐type boundaries parallel to (111)_c_ planes, and F‐type boundaries perpendicular to (111)_c_ planes. Among them, boundaries of C, D, E, F‐types have been reported in previous studies, with E and F‐type exhibiting atomic perturbations and high‐energy boundary [11, 16, 28].

In contrast to these earlier reports, our HAADF‐STEM combined with strain mapping analyses reveals that all boundaries are entirely natural and exhibit no significant strain (Figure 5). Notably, while no strain is detected at the boundaries, significant internal lattice strain is observed within the ordered domains, the values of which depends on the ordered arrangement of B‐site cations. Precise strain mapping of the 1:2 B‐site order superstructure reveals compressive strain for the B^I^‐site and tensile strain at the B^II^‐site, with maximal strains reaching up to 6% and ‐6%. This is further validated by LAADF‐STEM and Geometric Phase Analysis (GPA) mapping analyses in Figure S4. This suggests that lattice strain is accommodated within the ordered domains rather than at the boundaries.

To validate the magnitude of the ±6% intradomain lattice strain, we compared our findings with documented internal lattice strain levels in other perovskite oxides. For example, in BiFeO_3_, the out‐of‐plane lattice parameter near the R‐like/T‐like phase boundary varies by nearly 13% across fewer than ten‐unit cells, notably without obvious dislocations or defects [29]. In PIN‐PMN‐PT perovskite ferroelectric oxide micropillars, researchers have reported elastic strains of approximately 6%, with maximum elastic strains under compression surpassing 6% and total bending strains reaching 8.2% [30]. Similarly, freestanding single‐crystalline BiFeO_3_ membranes can withstand a maximum bending strain of 5.42% [31]. These precedents confirm that the ±6% intradomain strain observed in our study is physically sound and well within the expected range for perovskite oxides.

### 3.2 Atomic‐Scale Chemical Ordering

**FIGURE 6:**
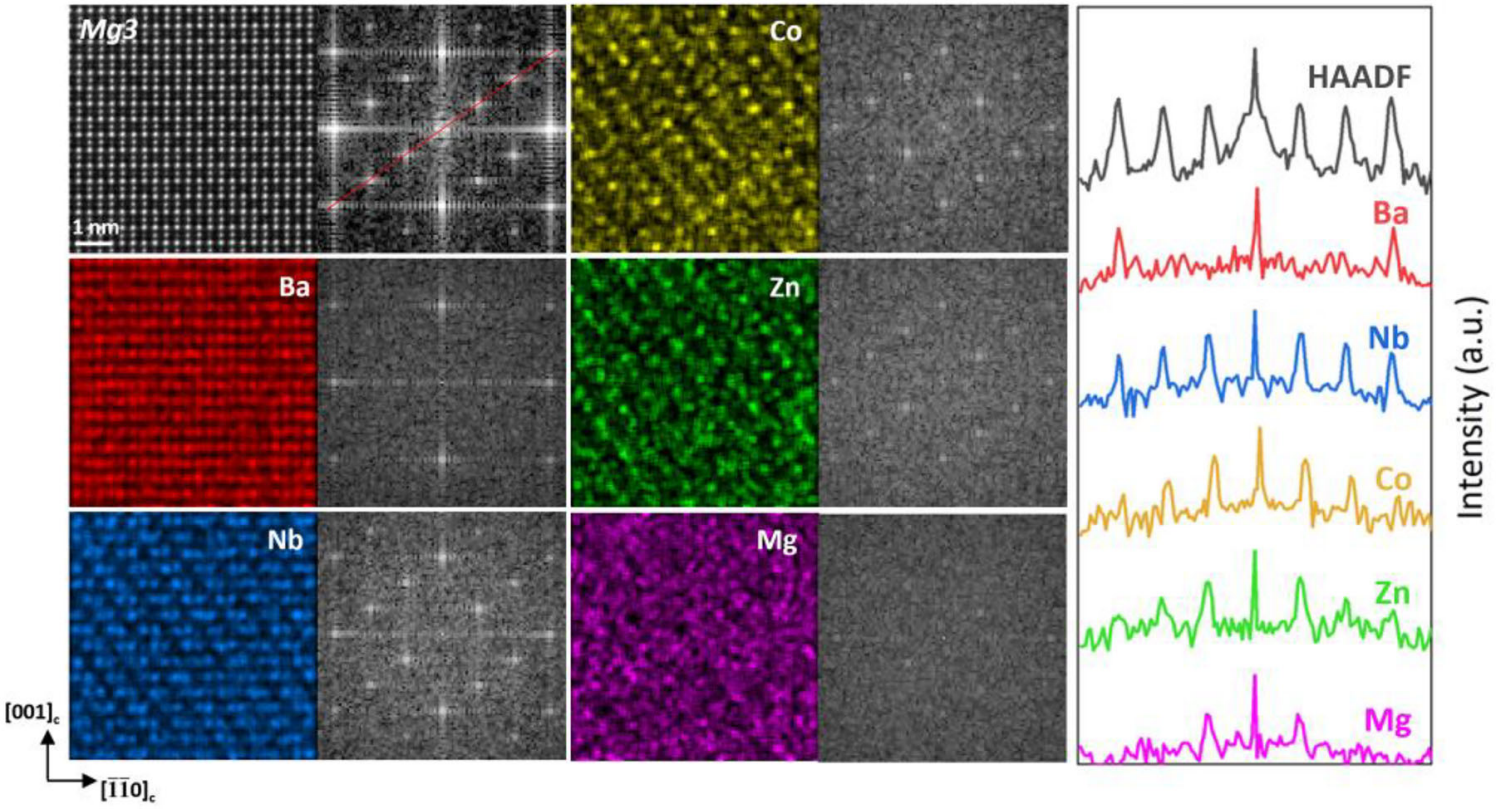
Atomic‐scale chemical distribution and cation ordering. Atomic‐scale HAADF‐STEM image, corresponding EDS element maps, FFT pattern of these elemental maps, and superlattice intensity profiles (extracted along the red line). These analyses reveal that the Co/Zn atoms and Nb atoms exhibit a higher degree of 1:2 ordering, whereas the Mg atoms display partial ordering.

The atomic‐scale EDS elemental analysis of the ordered domains in Mg3‐1475 ceramic provides important insights into the local ordering feature of B‐site cations, as shown in Figure 6, revealing irregularities of atomic arrangements within the ordered structure. Specifically, the weak (1/3, 1/3, 1/3) reflection in the FFT pattern of Mg EDS maps indicates partial order or positional variability of Mg atoms at the B‐site. In contrast, stronger (1/3, 1/3, 1/3) reflections in the FFT patterns of Co and Zn EDS maps suggest they mainly occupy the B^I^‐site. Thus, the 1:2 atomic arrangement in Mg3‐1475 is not perfect and does not strictly adhere to an ordered structure. In other words, Co, Zn, and Nb exhibit higher ordering degree in the 1:2 arrangement, while Mg atoms show disorder in their positions.

### 3.3 Oxygen Vacancy and Defect Analysis

**FIGURE 7:**
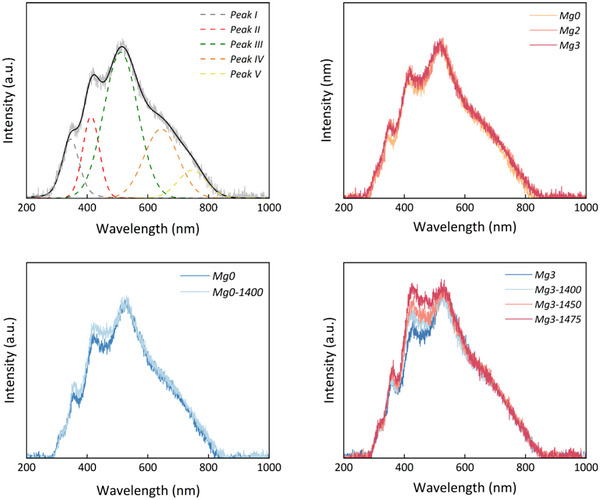
Oxygen vacancy and defect analysis. Ceramics with different Mg concentrations and annealing temperatures exhibit similar CL features, along with strong variations in the intensities associated with oxygen vacancies.

Defects, particularly oxygen vacancies, critically influence the structural evolution and physical properties of perovskite oxides. We carefully examined their presence and variation in Ba[(Co_0.6‐_ *_x_*_/2_Zn_0.4‐_ *_x_*_/2_Mg*_x_*)_1/3_Nb_2/3_]O_3_ ceramics. To quantitatively assess the oxygen vacancy concentration, we employed CL spectroscopy to analyze the defects, as this technique effectively probes variations in oxygen vacancy concentrations [32]. Figure 7 displays the CL spectra of the different samples. Peak *I* is centered at a wavelength of 345 nm, which corresponds to the indirect energy gap of BaNbO_3_ (∼3.6 eV). Peak *II* at 420 nm corresponds to a luminous transition of 2.9 eV, resulting from the transition of ionized oxygen vacancies to the valence band. Peak *III* at 517 nm originates from defective Nb–O_6_ octahedrons. Peak *IV* and Peak *V* at 646 nm and 746 nm arise from transitions at 1.9 eV and 1.4 eV, respectively, and are associated with strong luminescence from A‐site vacancies [32, 33, 34, 35, 36]. The results indicate that while an increase in Mg content does not significantly alter the oxygen vacancy concentration, increasing the annealing temperature leads to a substantial increase via thermal excitation.

### 3.4 Structure‐Property Correlation

**FIGURE 8:**
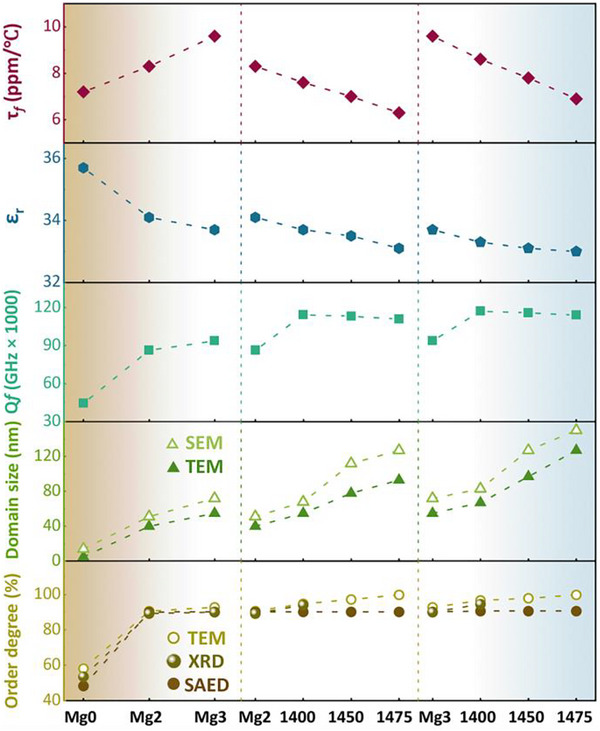
Evolution of structural and dielectric properties with Mg concentrations and annealing temperatures. Degree of ordering, domain size, quality factor Q*f*, dielectric constants *ε_r_*, and temperature coefficient of resonance frequency *τ_f_* of Ba[(Co_0.6‐x/2_Zn_0.4‐x/2_Mg_x_)_1/3_Nb_2/3_]O_3_ ceramics (x = 0, 0.2, 0.3) as a function of Mg concentrations and annealing temperature.

Figure 8 illustrates a general relationship among domain size, ordering degree, quality factor *Qf*, dielectric constants *ε_r_*, and temperature coefficient of resonance frequency *τ_f_*, suggesting a strong correlation between these parameters. To understand the underlying mechanisms, we further examined the microstructural origins of these properties. In this context, a kinetic analysis is highly valuable for elucidating the mechanisms by which Mg substitution and post‐sintering annealing promote domain growth. This analysis follows the classical grain/domain growth relation:

(1) $$D^{n}-{D_{0}}^{n}=\mathrm{Kt}$$

where *D* is the average ordered domain size after annealing for time *t*, *D*_0_ is the initial ordered domain size, *n* is the growth exponent, and *K* is the temperature‐dependent growth rate constant, the temperature dependence of which is described by the classical Arrhenius equation [37, 38]:

(2) $$K=K_{0}\;e^{-\frac{E_{a}}{RT}}$$

where *K*_0_ is the pre‐exponential factor, *E_a_* is the intrinsic activation energy of the coarsening process, *R* is the ideal gas constant (8.314 J mol^−1^·K^−1^), and *T* is the absolute temperature. Because complete time‐temperature coarsening evolution curves are unavailable to strictly determine the growth exponent *n*, we assume *n* = 1 in this semi‐quantitative estimate. On this basis, the temperature‐dependent domain‐size data were linearly fitted using the Arrhenius‐type relation:

(3) $$\ln\left(D-D_{0}\right)=\ln\left(\;K_{0}t\right)-\frac{E_{a,app}}{RT}$$

where E*_a, app_* is the energy term extracted from the slope was defined as the apparent activation energy for ordered‐domain growth, rather than the intrinsic diffusion activation energy of any specific B‐site cation. To extract the apparent activation energy from the experimental data, ln ((*D* − *D*_0_)/*nm*) is plotted as the ordinate and 1000/*T* as the abscissa, followed by linear fitting, with the temperature‐independent terms combined into a single constant C, the fitting equation above can be equivalently transformed:

(4) $$\ln\left(\left(D-D_{0}\right)/nm\right)=C-\left(\frac{E_{a,app}}{1000R}\right)\left(\frac{1000}{T}\right)$$

where the post‐annealing duration for all samples in Table 1 is fixed at 12 hours, *D* is the TEM‐derived domain size after post‐annealing, *D*_0_ is the initial domain size (40 nm for Mg2 and 55 nm for Mg3). Based on the TEM domain sizes of 55 nm, 78 nm, and 93 nm for the Mg2‐1400, Mg2‐1450, and Mg2‐1475 samples, respectively, and an initial size *D*_0_ = 40 nm, *E_a, app_* is calculated to be 414.8 kJ mol^−1^ (4.30 eV). For the Mg3 samples (sizes of 67 nm, 97 nm, and 127 nm) with *D*_0_ = 55 nm, *E_a, app_* is determined to be 584.0 kJ mol^−1^ (6.05 eV). The Mg0 sample was not fitted as only one post‐annealing temperature point is available. These results indicate that the coarsening of domains during post‐annealing exhibits distinct apparent thermally activated characteristics, and apparent activation energy increases with Mg content, as shown in Figure S5.

Furthermore, we identified a two‐stage ordering mechanism: in the initial stage of domain growth (where B‐site cations exhibit 1:2 ordering), the ordering degree increases with domain size, accompanied by enhanced dielectric *Qf* value; however, once ordered domains mostly cover the grain volume, the 1:2 B‐site arrangement within the domains becomes disrupted by Mg disorder, leading to a decrease in ordering degree and consequent degradation of *Qf* value. Moreover, we consider the synergistic effects of Mg disorder, oxygen vacancies, and intradomain strain. Under high‐temperature annealing conditions, although the domains undergo sufficient growth, thermal excitation simultaneously generates a massive number of oxygen vacancies. These high‐concentration oxygen vacancies disrupt the periodic potential field of the lattice, acting as additional phonon scattering centers and thereby significantly intensifying the phonon scattering processes [39]. In addition, the incomplete ordering of Mg degrades the high macroscopic symmetry of the lattice, inducing extra phonon scattering and subsequently leading to a pronounced increase in the damping of polar optical phonons [40, 41]. Coupled with a local lattice strain field of up to ±6% within the domains [39, 42], these three factors collectively disrupt the translational symmetry of the lattice. They act as exceptionally strong phonon scattering centers that significantly shorten the phonon lifetime, ultimately leading to the deterioration of the *Qf* value.

Regarding the impact on *ε_r_* and *τ_f_*, intradomain strain is known to modulate these parameters by altering ionic polarizability, lattice vibrational modes, and lattice anharmonicity [39, 43]. Specifically, local tensile or compressive strain can modify the B–O bond lengths and B─O─B bond angles, thereby affecting local ionic displacements and polarization strengths [44, 45]. Furthermore, the interplay of strain fields, Mg disorder, and oxygen vacancies can disrupt the long‐range periodicity of the lattice. This disruption generates local random electric fields or short‐range polar structures, subsequently introducing additional dielectric relaxation processes [46, 47, 48]. Given that the strain distribution in our system is spatially nonuniform and coupled with defect concentrations, isolating its exact quantitative contribution to *ε_r_* and *τ_f_* from the overall dielectric response is challenging. However, we can qualitatively conclude that the enhanced phonon scattering driven by intradomain lattice strain not only exacerbates microwave dielectric loss but also influences the frequency and temperature stability of the dielectric response.

Beyond these strain and defect‐related mechanisms, variations in Mg content and annealing temperature further modulate *ε_r_* and τ_ƒ_. With increasing Mg concentrations, the dielectric constant decreases, primarily attributed to the lower ion polarization of Mg^2+^ compared to Co^2+^ and Zn^2+^ ions. After annealing, the dielectric constants of each component in the sample slightly decrease. This is because annealing increases the ordering degree of the sample, resulting in enhanced covalence and weakened polarization, leading to a decrease in dielectric constants. In addition, Ba(Mg_1/3_Nb_2/3_)O_3_ owns a higher temperature coefficient of resonant frequency τ_ƒ_ than Ba(Zn_1/3_Nb_2/3_)O_3_ and Ba(Co_1/3_Nb_2/3_)O_3_, when Ba[(Co, Zn, Mg)_1/3_Nb_2/3_]O_3_ is formed. The τ_ƒ_ increases gradually with the increase of Mg^2+^, but remains less than 10 ppm/°C [39].

Future work should focus on the regulation of chemical ordering and lattice strain, specifically, First, regarding lattice‐strain management, one potential approach is targeted strain compensation via the introduction of B‐site cations with appreciably different ionic radii, such as Zr^4+^ [12, 49]. Partial substitution at the Nb site could modulate the local lattice stress distribution. Ideally, this would reduce the intradomain strain (which currently reaches up to ±6%) and mitigate the associated excessive phonon scattering, all without disrupting the 1:2 ordered structure. Such a strategy holds promise for optimizing microwave dielectric loss while preserving a high ordering degree. Second, to mitigate point defects generated during high‐temperature annealing, we propose exploring rapid quenching or high‐oxygen‐pressure sintering techniques. These processes can help suppress the concentration and aggregation of thermally activated defects, particularly oxygen vacancies [50, 51, 52]. By carefully coordinating the oxygen partial pressure and cooling rate, it may be possible to realize a microstructure that features both large domains and a low defect density. This would offer a highly practical pathway toward next‐generation high‐performance microwave dielectric materials.

## 4 Conclusions

In summary, this work establishes a multiscale characterization methodology to evaluate the domain size and ordering degree and to analyze the domain evolution process in complex perovskite oxide ceramics. By systematically correlating results from the micrometer to the atomic scale, we elucidated the competitive relationship between order and disorder and its direct impact on microwave dielectric properties. We identified a two‐stage ordering mechanism: initially, the average ordering degree alters with domain coarsening; however, once domains mostly cover the grain volume, the 1:2 B‐site arrangement becomes disrupted by Mg disorder. This Mg disorder, together with the oxygen vacancies generated by thermal excitation and the intradomain strain arising from the 1:2 ordering of B‐site cations, collectively disrupts the translational symmetry of the lattice. This creates strong phonon scattering centers that shorten the phonon lifetime, ultimately leading to degradation of the *Qf* value. These findings provide a new perspective on the structure‐property relationship in multi‐component perovskite oxide materials, and suggest that future optimization of their dielectric performance may focus on manipulating chemical ordering and lattice strain within domains, rather than solely altering domain sizes.

## 5 Experimental

A series of Ba[(Co_0.6‐x/2_Zn_0.4‐x/2_Mg_x_)_1/3_Nb_2/3_]O_3_ ceramics (with *x* = 0, 0.2 and 0.3) were prepared stoichiometrically via a conventional solid‐state reaction method. Reagent‐grade powders of BaCO_3_ (99.93%), CoO (99.9%), ZnO (99.95%), MgO (99.9%), and Nb_2_O_5_ (99.99%) were used as starting materials. The samples were sintered in air between 1425 °C and 1575 °C for 3 h (these as‐sintered samples are hereafter labelled as Mg0, Mg2 and Mg3, respectively). Subsequently, some of the as‐sintered samples were annealed in air for 12 h at 1400 °C, 1450 °C, and 1475 °C, respectively. These are labeled with the suffix of their annealing (for example, annealed Mg3 samples are named as Mg3‐1400, Mg3‐1450 and Mg3‐1475, respectively).

Microstructural characterization was performed on polished surfaces using a field‐emission scanning electron microscope (SEM; model Gemini G300, Carl Zeiss, Germany). Elemental analyses were performed using an energy‐dispersive X‐ray spectrometer (EDS; model X‐Max 51, Oxford Instruments, UK) attached to this SEM. Luminous defects were investigated by cathodoluminescence (CL) spectra collected with an optical spectrometer (model iHR 320, Horiba Jobin Yvon, Japan), which is also attached with the same SEM. Electron channeling contrast imaging (ECCI) was utilized to visualize domains at the sub‐micron scale. Nanoscale domain structures were investigated on a field‐emission transmission electron microscope (model JEM‐F200, JEOL, Japan) operated at 200 kV. Atomic‐scale scanning transmission electron microscopy (STEM) observations and elemental analyses of domains were conducted on a double aberration‐corrected TEM (model Spectra 300, ThermoFisher, USA) operated at 300 kV, equipped with a Super‐X EDS detector. High‐angle annular dark‐field (HAADF) images and low‐angle annular dark‐field (LAADF) images were obtained using a collection semi‐angle of 62–200 mrad and 30–59 mrad, respectively, and a convergence semi‐angle of 26.0 mrad. Convergent beam electron diffraction (CBED) was performed with a convergence angle of 10.0 mrad.

Microwave dielectric properties were measured using a vector network analyzer (Agilent 8753ES, Agilent Technologies Inc., Santa Clara, CA). The quality factor *Q* (inverse of the dielectric loss, tan δ) was determined via the resonant‐cavity method at 4–6 GHz. Because the *Q* factor generally varies inversely with the frequency, in the microwave region, the product of *Qf* was used to evaluate the dielectric loss instead of *Q*. Dielectric constant *ε_r_* and temperature coefficient of resonance frequency *τ_f_* were evaluated by Hakki–Coleman method. Resonant frequency data at the temperature of 20 °C–80 °C were collected, and *τ_f_* was calculated according to *τ_f_*_=_ $\frac{f_{80}-f_{20}}{f_{20}\;\times \;\left(80-20\right)}$, where *f*_20_ and *f*_80_ are the resonance frequency at 20 °C and 80 °C, respectively. These methods for properties all refer to references [15, 16].

## Author Contributions

**Hui Gu**: conceptualization, Writing – review and editing, methodology, resources, supervision, investigation, formal analysis, data curation, project administration. **Pianpian Ma**: data curation, resources. **Xiangming Chen**: resources, investigation. **Qingqiao Fu**: Writing – original draft, conceptualization, software, data curation, investigation, validation, funding acquisition, visualization, methodology, Writing – review and editing. **Qiang Zheng**: data curation, Writing – review and editing, methodology, software, supervision, validation, visualization, conceptualization, project administration. **Hanbin Gao**: software, data curation, validation. **Juanjuan Xing**: Writing – review and editing, formal analysis, funding acquisition, supervision.

## Conflicts of Interest

The authors declare no conflicts of interest.

## Supporting information




**Supporting File**: advs76785‐sup‐0001‐SuppMat.docx.

## Data Availability

The data that support the findings of this study are available on request from the corresponding author. The data are not publicly available due to privacy or ethical restrictions.
