# Supplementary material for: Unraveling Hierarchical B‐Site Ordering: Mechanism of Microwave Dielectric Enhancement in Complex Perovskite Oxide Ceramics
Source: Adv Sci (Weinh). 2026 Jul 29:e76785. Online ahead of print. doi: 10.1002/advs.76785 (PMC13418049; doi:10.1002/advs.76785)
Supplement: Supplementary file 1 — Supporting File: advs76785‐sup‐0001‐SuppMat.docx. [file ADVS-9999-e76785-s001.docx]

Supporting Information for

**Unraveling Hierarchical B-Site Ordering: Mechanism of Microwave Dielectric Enhancement in Complex Perovskite Oxide Ceramics**

*Qingqiao Fu, Hui Gu,^*^ Hanbin Gao, Pianpian Ma, Xiangming Chen, Juanjuan Xing,^*^ Qiang Zheng^*^*

Q.Q. Fu, J.J. Xing

School of Materials Science and Engineering, Shanghai University, Shanghai 200444, China

E-mail: [xingjuanjuan@shu.edu.cn](mailto:xingjuanjuan@shu.edu.cn)

H. Gu

Center for High Pressure Science & Technology Advanced Research, Beijing 100193, China

E-mail: [hui.gu@hpstar.ac.cn](mailto:hui.gu@hpstar.ac.cn)

H.B. Gao, Q. Zheng

CAS Key Laboratory of Standardization and Measurement for Nanotechnology, National Center for Nanoscience and Technology, Beijing 100190, China

E-mail: [zhengq@nanoctr.cn](mailto:zhengq@nanoctr.cn)

P.P. Ma

School of Materials Science and Engineering, Zhejiang Sci-Tech University, Hangzhou 310018, China

X.M. Chen

School of Materials Science and Engineering, Zhejiang University, Hangzhou, 310027, China


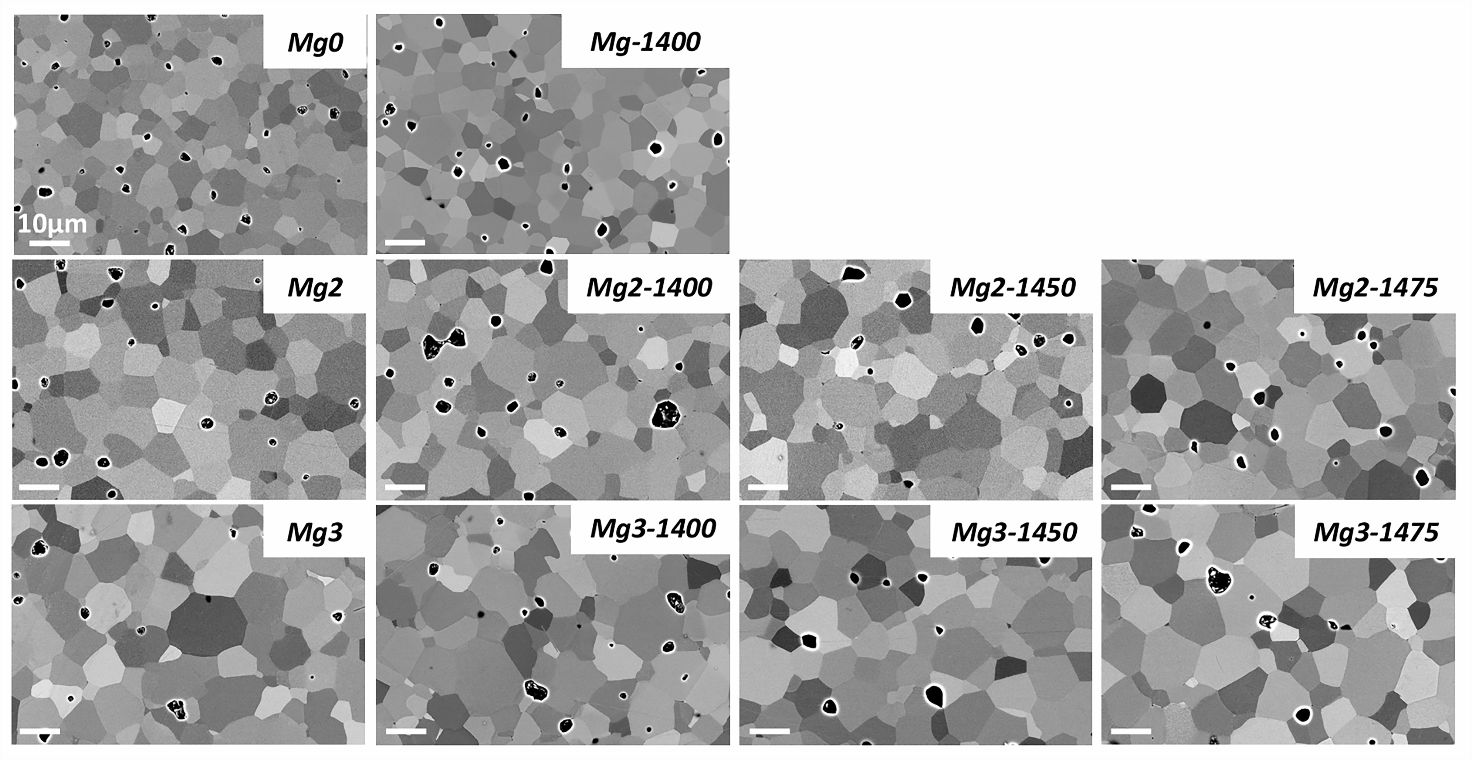


**Figure S1** Low magnification SEM-BSE morphology images of all ceramics.


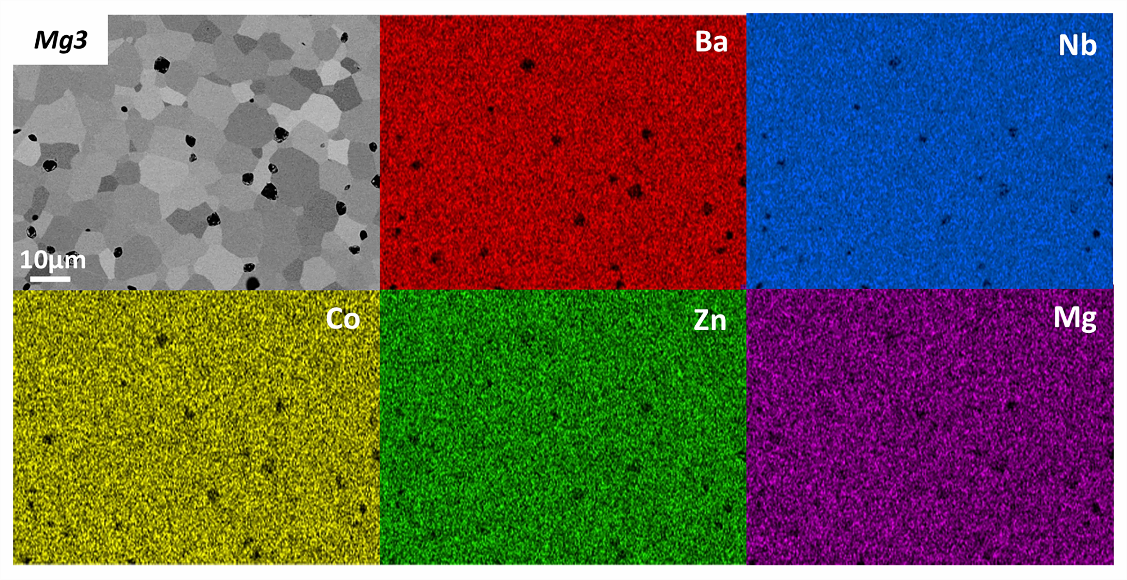


**Figure S2** SEM-BSE image and EDS elemental mapping of Mg3 ceramic present a homogeneous composition without secondary phase.


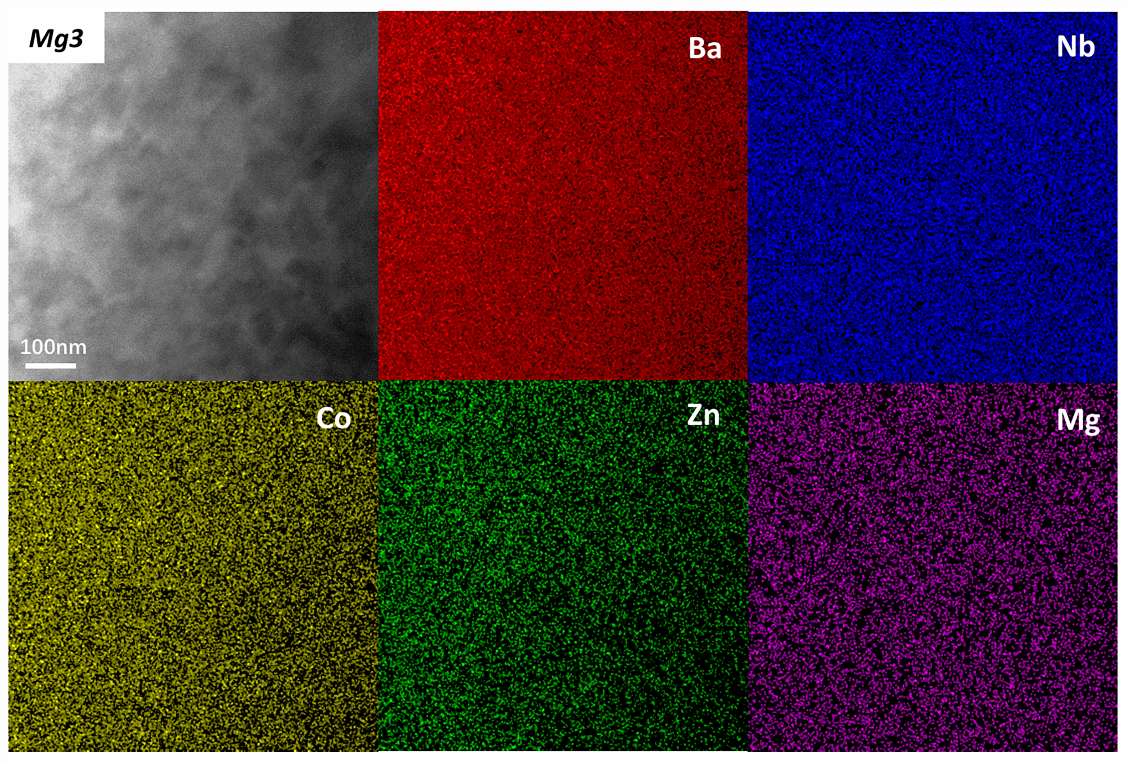


**Figure S3** EDS elemental mapping of Mg3 grain exhibit uniform chemical composition for domains in different orientations.


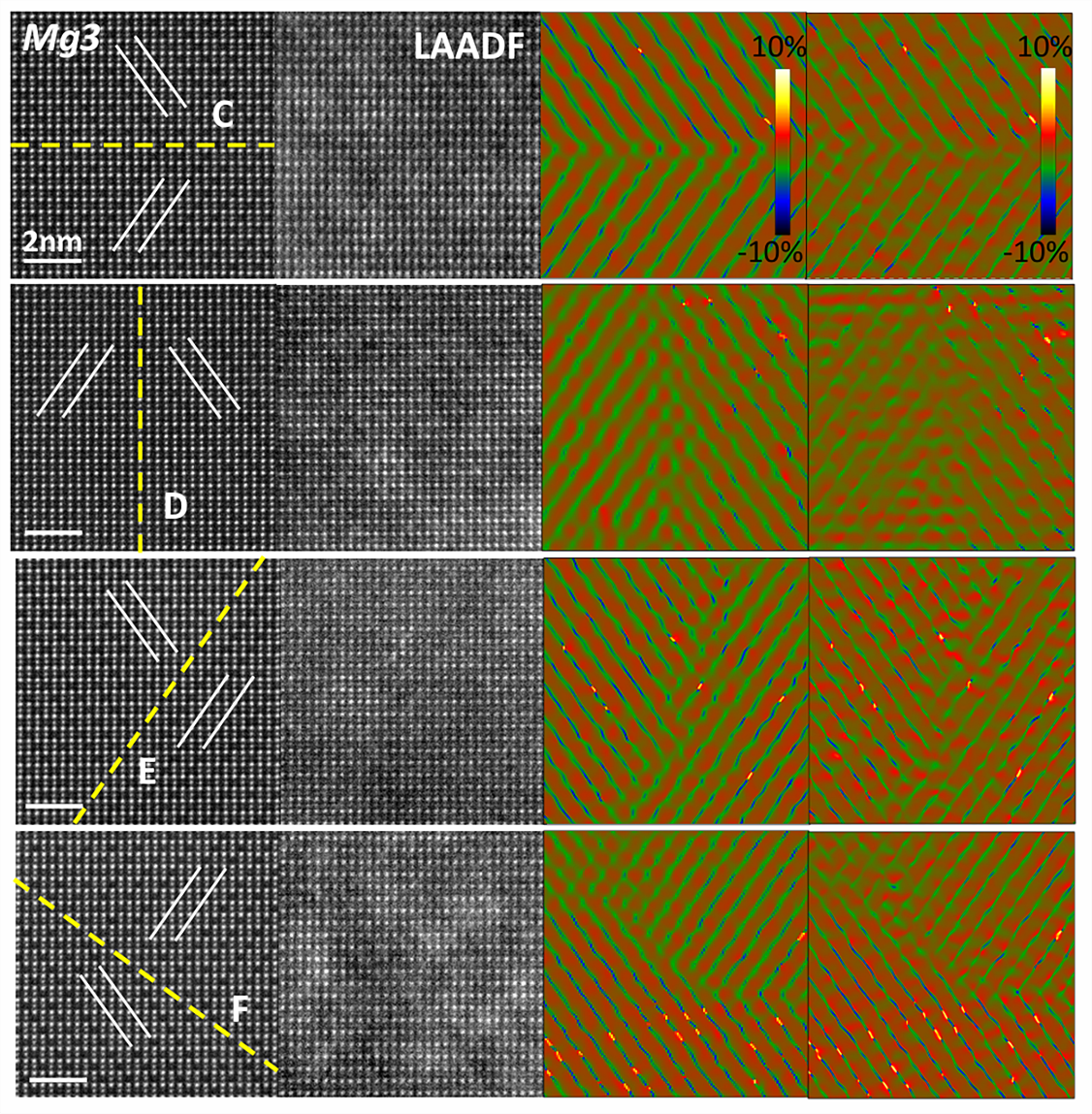


**Figure S4** LAADF-STEM and GPA mapping analyses reveal natural transitions in domain boundaries, without significant strain. However, the strain within the domains varies based on the 1:2 B-site ordered arrangement.


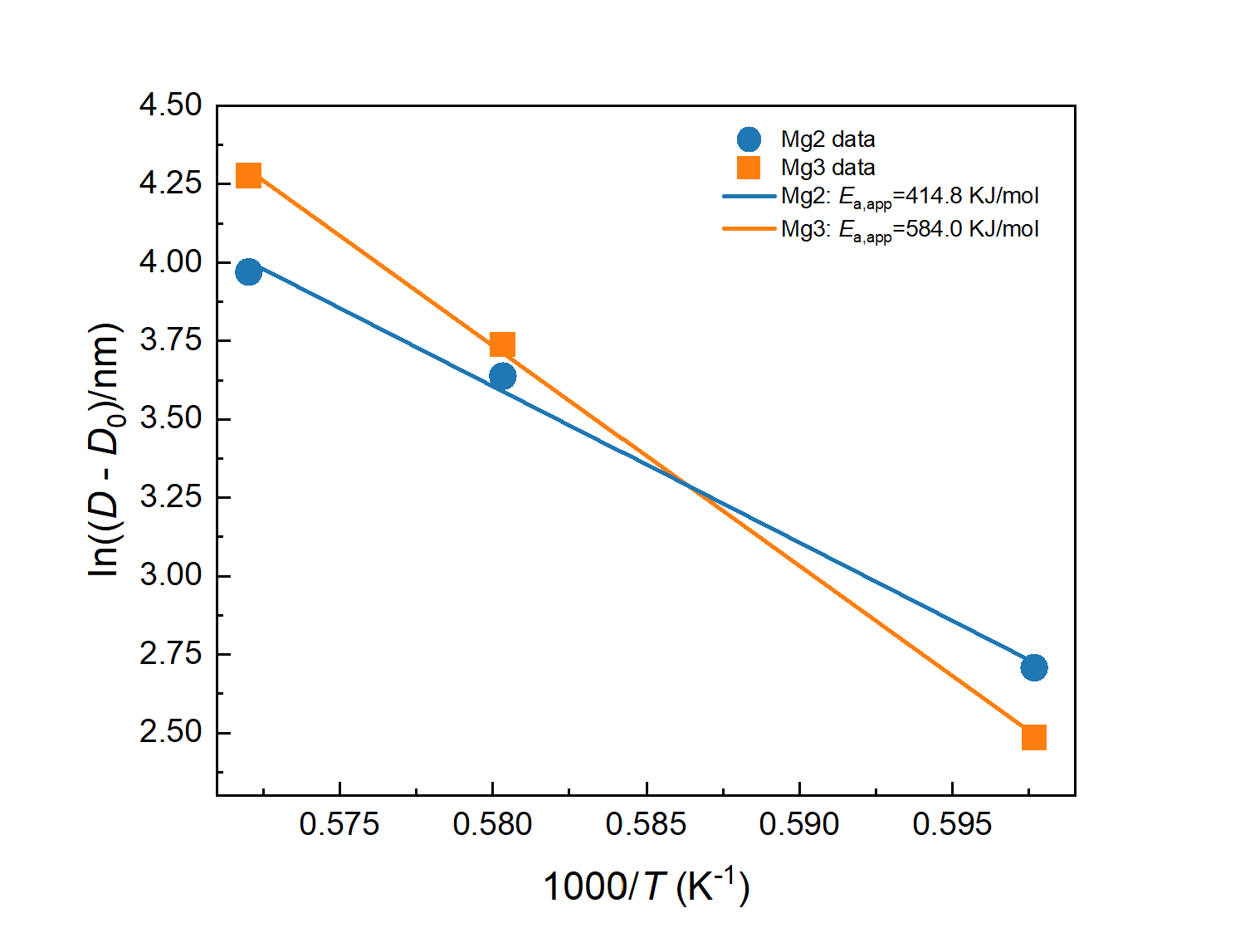


**Figure S5** Arrhenius-type plots for estimating the apparent activation energies (*E_a,app_*) of ordered domain growth in the Mg2 and Mg3 samples.
